# MicroRNA-206 Regulation of Skin Pigmentation in Koi Carp (*Cyprinus carpio* L.)

**DOI:** 10.3389/fgene.2020.00047

**Published:** 2020-02-12

**Authors:** Zaijie Dong, Mingkun Luo, Lanmei Wang, Haoran Yin, Wenbin Zhu, Jianjun Fu

**Affiliations:** ^1^ Key Laboratory of Freshwater Fisheries and Germplasm Resources Utilization, Freshwater Fisheries Research Center of Chinese Academy of Fishery Sciences, Ministry of Agriculture and Rural Affairs, Jiangsu, China; ^2^ Wuxi Fisheries College, Nanjing Agricultural University, Jiangsu, China

**Keywords:** MiRNA-206, *Mc1r*, skin color, melanocytes, koi carp

## Abstract

MicroRNAs (miRNAs) are ∼22 nucleotide non-coding RNA molecules that act as crucial roles in plenty of biological processes. However, the molecular and cellular mechanisms of miRNAs to regulate skin color differentiation and pigmentation in fish have not been fully understood. Herein, we revealed that miR-206, a skin-enriched miRNA, regulates melanocortin 1 receptor (*Mc1r*, a key regulator of melanogenesis) expression by binding to its 3'-untranslated (UTR) region through bioinformatics and luciferase reporter assay in koi carp (*Cyprinus carpio* L.). The analysis of spatial and temporal expression patterns suggested that miR-206 is a potential regulator in the skin pigmentation process. Then, we silenced it *in vivo* with an antagomir method. The result showed a substantial increase of *Mc1r* mRNA expression and protein level, and also its downstream genes: tyrosinase (*Tyr*) and dopachrome tautomerase (*Dct*) that encoding key enzymes involved in melanin synthesis. Moreover, we constructed the miRNA-206 sponge lentivirus vector to transfect koi carp melanocytes *in vitro*, further checked the functions of melanocytes using Cck-8 and Transwell assays. As a result, inhibition of miR-206 significantly up-regulated *Mc1r* mRNA expression and protein level and accelerated the melanocyte proliferation and migration ability compared with the scrambled-sequence negative control group (miR-NC). Overall, these findings provide the evidence that miR-206 plays a regulatory role in the skin color pigmentation through targeting the *Mc1r* gene and would facilitate understanding the molecular regulatory mechanisms underlying miRNA-mediated skin color pigmentation in koi carp.

## Introduction

MicroRNAs (miRNAs) are single-stranded, evolutionary conserved ~22 nucleotides (nt) molecules that affect the gene expression by triggering either mRNA degradation or translational repression through combining the 3'-untranslated regions (3'-UTR) (Ambros, 2004; Krol et al., 2010). Over the past years, the researches about the interaction between miRNAs and mRNAs have gradually verified that it is crucial for transcriptional and signal transduction events like development, differentiation, cell proliferation, and apoptosis (Hwang and Mendell, 2006; Yan et al., 2013; Wang et al., 2017). Meanwhile, the accumulating evidence reveals that miRNAs also play an essential role in animal skin. For example, the skin color of the mouse changes from black to brown when over-expression of miR-137 (Dong et al., 2012). Mir-203 was identified as a molecular switch between the proliferative basal progenitors and terminally differentiating suprabasal cells that ensure the proper identity of neighboring layers (Yi et al., 2008). The dorsal/abdomen pigmentation was substantially decreased upon miR-8 lost in *Drosophila* (Kennell et al., 2012). Yan et al. identified the miR-429 act as a crucial role in skin pigmentation by targeting *Foxd3* 3'-untranslated (3'-UTR) region and silencing its expression *in vivo* would repress the transcription level of *Mitf* and its downstream genes (Yan et al., 2013). In our previous study, we have identified 164 differentially expressed miRNAs (DEMs) and 14 overlapping including miR-206 on different skin colors (black, red, and white) through Illumina sequencing in koi carp (Luo et al., 2018). However, miRNAs specific regulation mechanisms related to skin color differentiation in koi carp remains unclear.

Skin colors and their different patterns are important phenotypes that suitable for fitness under the natural environment for species (Protas and Patel, 2008). So far, six types of pigment cells (i.e. melanocytes, xanthophores, erythrophores, iridophores, leucophores, and cyanophores) have been reported in fish, which provided excellent materials for the study of biological development and evolutionary applications (Kelsh, 2004; Braasch et al., 2007; Ceol et al., 2008). Some studies have identified that the ratio of eumelanin and pheomelanin largely determines an animal's overall color, in which, eumelanin is responsible for black to brown color and the other is responsible for red to yellow (McGraw et al., 2005). In melanocytes, two primary genes, i.e. melanocortin 1 receptor (*Mc1r*) and agouti signaling protein (*Asip*), act as two switches in the melanogenesis pathway. The levels of *Mc1r* activity makes intracellular cyclic AMP (cAMP) sufficiently high to activate the eumelanin synthetic when the *Asip* protein is absent, while the *Mc1r* activity is inhibited and starts producing pheomelanin when the *Asip* protein is presence (Hoekstra, 2006). Additionally, a handful of genes known to alter the density and distribution of melanosomes involved in the melanogenesis pathway have also been identified. Tyrosinase (*Tyr)*, for example, is an important enzyme in eumelanin synthesis that its activation can be stimulated to produce more melanin when the expression of microphthalmia-associated transcription factor (*Mitf*) is increased (Altschmied et al., 2002). Other genes, such as tyrosinase-related protein-1 (*Tyrp1*) and dopachrome tautomerase (*Dct* or *Tyrp2*), primarily regulate the eumelanin pathway (Braasch et al., 2009). Interestingly, we also identified some important miRNAs and mRNAs on black, white, and red skin of koi carp, which involved in the cAMP signaling, melanogenesis, and tyrosine metabolism pathway through Illumina sequencing (Luo et al., 2018; Luo et al., 2019). Therefore, it is necessary to further excavate the specific relationship between the coding and non-coding RNAs, e.g. *Mc1r* gene and its target miRNAs, at the molecular, genetic, and developmental levels of skin pigmentation in koi carp.

Koi carp, a colorful variety of common carp (*Cyprinus carpio*), is one of the popular ornamental fish spread worldwide that distinguished by coloration, patterning, and scalation (Damme et al., 2010). Extensive studies have been conducted on skin color regulation, including rearing environment (Jha and Barat, 2006), feed additives (Lin et al., 2011; Manas et al., 2017), genetics of pigment patterning (Wang and Li, 2004; Chistiakov and Voronova, 2009), gene functional analysis (Bar et al., 2013; Liu et al., 2015), and transcriptome analysis (Xu et al., 2014). Among these complicated regulation factors, genetics elements are the most important factors, thus the majority of gene resources related to skin color pigmentation need to be further elucidated. In this study, we excavated the potential function of miR-206 in koi carp skin color. First, we identified the *Mc1r* gene, involved in the melanogenesis pathway, as a target mRNA of miR-206 through a bioinformatics method. Then, we validated the crosstalk between miR-206 and *Mc1r* mRNA by qRT-PCR and luciferase reporter assays. Moreover, we silenced miR-206 *in vivo* with antagomir to explore the function. Finally, we further analyzed the interaction between miR-206 and *Mc1r* in melanocytes through qRT-PCR, western-blot, cck-8, and transwell assays. Our results revealed that miR-206 might play a significant role in skin color differentiation and pigmentation in koi carp.

## Results

### Prediction of MiRNAs Targeting *Mc1r*

**Figure 1:**
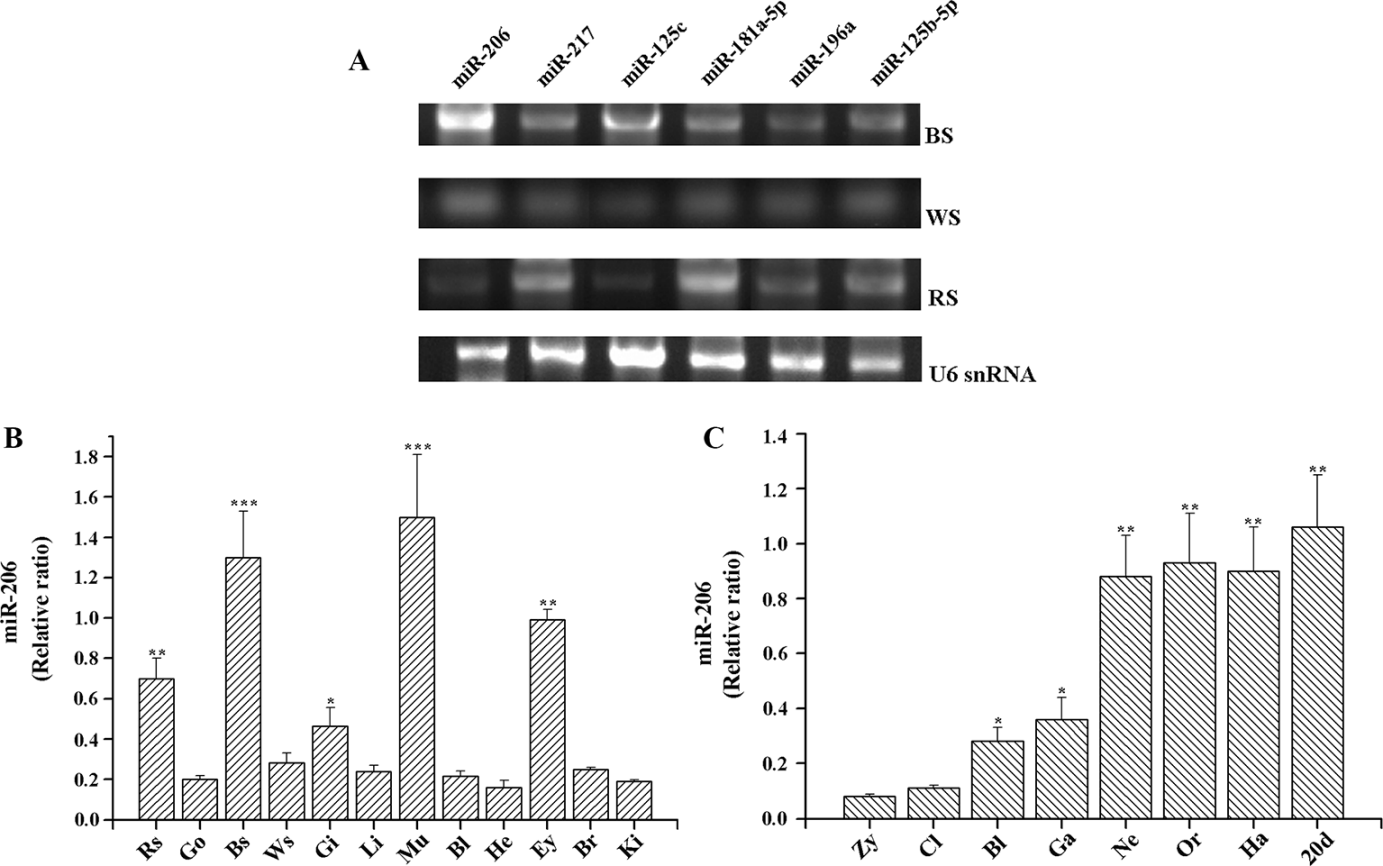
Expression profiles of miRNAs in koi carp. **(A)** Expression patterns of six miRNAs in three skin color samples. MiRNA samples were extracted from the different tissues, including BS (black skin), WS (white skin), and RS (red skin). MiRNAs expression levels were detected by RT-PCR. *U6* snRNA was detected as the loading control. **(B)** Expression patterns of miR-206 in different tissues. Rs, red skin; Go, gonad; Bs, black skin; Ws, white skin; Gi, gill; Li, liver; Mu, muscle; Bl, blood; He, heart; Ey, eye; Br, brain; Ki, kidney. **(C)** Overview of expression patterns during the early stages of koi carp. Zy, zygote; Cl, cleavage; Bl, blastula; Ga, gastrula; Ne, neurula; Or, organogenesis; Ha, hatching; 20 pdh, 20 days post-hatching. *p < 0.05, **p < 0.01, ***p < 0.001.

MiRNAs target identification is important to predict their functions, thus the strategies applied should reduce the number of false-positive miRNA targets (Bandyopadhyay and Mitra, 2009). In our previous study, we used a combination of TargetScan (http://www.targetscan.org/), RNAhybrid (https://omictools.com/rnahybrid-tool), and miRanda (https://omictools.com/miranda-tool) software to predict the skin color related-miRNA target genes by alignment with common carp (*C. carpio*) genome sequence, the final results must be consistent with the analysis of all three software in order to improve the accuracy (Additional file 3, **Table S2**). The results indicated that *Mc1r* may be regulated by more than one miRNA, including miR-206, miR-217, miR-125c, miR-181a-5p, miR-196a, and miR-125b-5p. Then, we detected their expression level among three different skin tissues. The results of transcripts per million (TPM) number showed that there were different expression patterns (**Table 1**). Additionally, we also conducted RT-PCR and qRT-PCR assays to investigate the expression patterns. Of these six miRNAs, miR-206 was expressed strongly in the black skin than the other five miRNAs (**Figure 1A**) and up-regulated in black skin than the other two skins (**Figure 1B**). Given that *Mc1r* was also found to be mainly involved in the melanogenesis signaling pathway, we thus explore whether there exists a relationship between miR-206 and *Mc1r* in the follow-up work.

**Table 1:** Differentially expressed miRNAs among three skin tissues.

MiR-name	WS-std	BS-std	Fold_change (Log2 BS/WS)	sig	RS-std	BS-std	Fold_change (Log2 BS/RS)	sig
miR-206	700.92	2065.05	1.56	**	400.21	2065.05	2.37	**
miR-217	37.47	76.48	1.03	**	51.31	76.48	0.58	*
miR-125c	0.01	40.74	11.99	**	0.01	40.74	11.99	**
miR-181a-5p	1.77	8.56	2.28	**	6.49	8.56	0.46	*
miR-196a	123.45	708.24	6.64	**	43.31	708.24	4.032	**
miR-125b-5p	0.01	3.59	8.488	**	0.01	3.59	8.488	**

* P < 0.05, ** P < 0.01.

### Expression Pattern of miR-206 in Koi Carp

To further gain insight into the function of miR-206 in physiological process, we also detected its temporal and spatial expression levels by qRT-PCR. The results indicated that miR-206 mainly expressed in black skin, muscle, eyes, and red skin (**Figure 1B**). Meanwhile, we detected its expression profile during eight different developmental stages in koi carp (**Figure 1C**). We found miR-206 represented a dynamic expression pattern, and the expression level was highly up-regulated in the neurula stage, then sustained through to the larvae stage (20 days post-hatching). In addition, the pigment cells are initially derived from the neural crest during the gastrula stage (Betancur et al., 2010), thus we guess miR-206 might act as a potential regulator involved in the skin pigmentation process.

### MiR-206 Acts Directly at the 3'UTR of *Mc1r*

**Figure 2:**
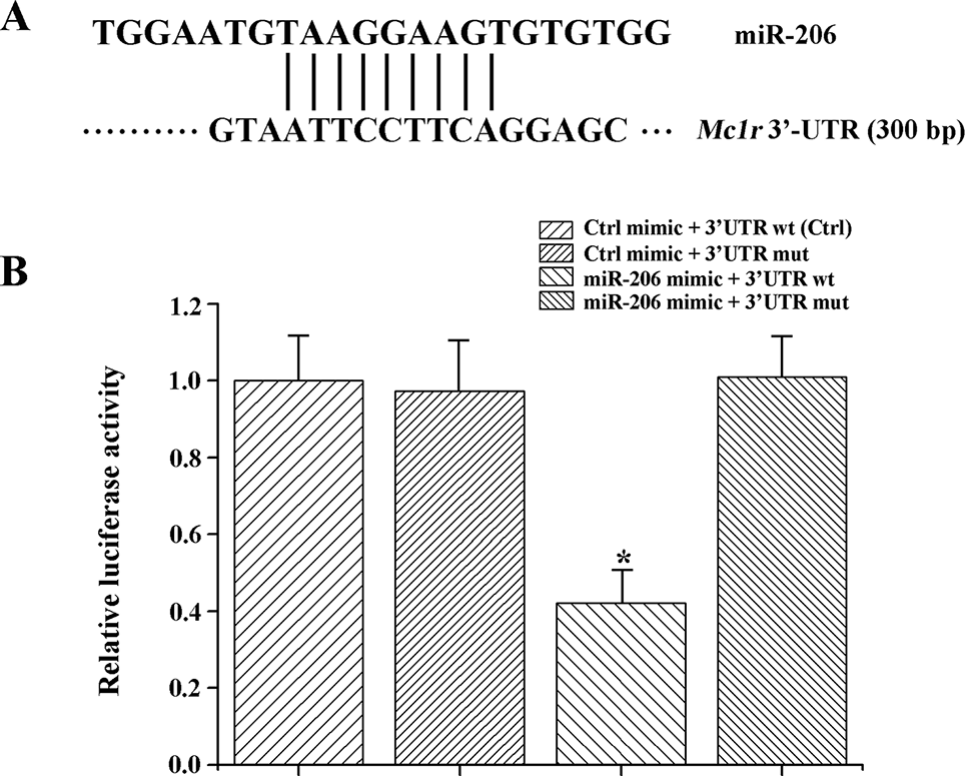
MiR-206 acts directly at the 3'-UTR of *Mc1r*. **(A)** The alignment between the miR-206 and 3'-UTR segment of *Mc1r*. **(B)** Dual luciferase reporter assay system was used to test binding between miR-206 and *Mc1r* in HEK293T cells. The treatment of control mimic + 3'-UTR wt in HEK293T cell lines was taken as the control group. *P < 0.05.

In animals, miRNA generally function through uninterrupted base-pairing between 2~7 nucleotide (seed sequence) and complementary sequence in the 3'-UTR of a target mRNA (Grimson et al., 2007). Herein, we found the *Mc1r* gene (Genbank No: JX989223.1, with full length of 1858 bp, coding for 321 amino acids and 300 bp 3'-UTR region) had a seed region of 3'-UTR completely matched (from 1,581 bp to 1,589 bp) the seed sequence (nine-nucleotide) of miR-206 (**Figure 2A**).

To further verify the interaction between miR-206 and *Mc1r*, we employed two luciferase reporters, which were the wild-type 3'-UTR of *Mc1r* gene, or the mutant UTR of the *Mc1r* gene. Then, the luciferase reporters were co-transfected with miR-206 mimic into HEK293T cells. And a scrambled miRNA mimic with no homology to the common carp genome was used to control. The results revealed that the miR-206 mimic significantly decreased the luciferase activity of wild-type *Mc1r* 3'UTR, while it did not affect the mutant *Mc1r* 3'UTR, suggesting that miR-206 directly suppressed *Mc1r* expression level by targeting its 3'UTR region (**Figure 2B**).

### Validation of the Interaction Between miR-206 and *Mc1r In Vivo*

**Figure 3:**
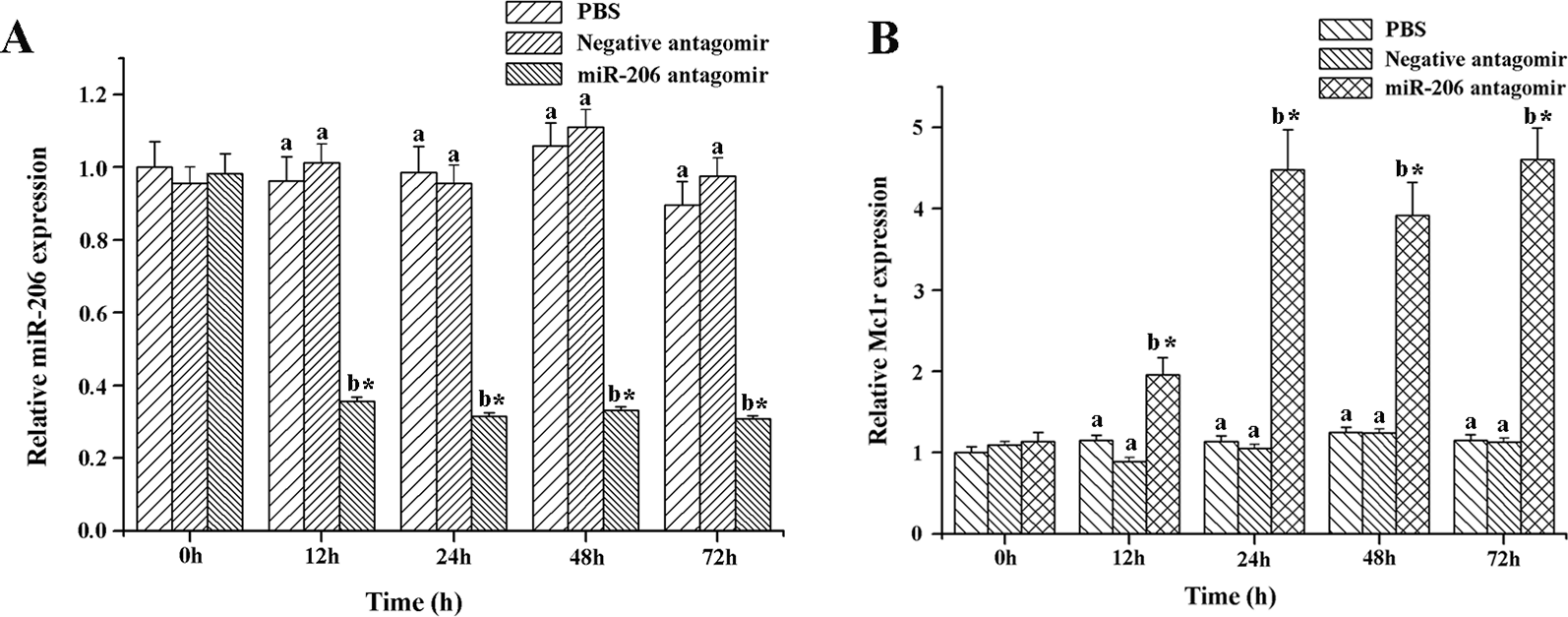
Effect of miR-206 silencing on expression of miR-206 **(A)** and *Mc1r* gene **(B)**. Koi carp weighing ~10g were received a tail-vein injection with PBS, negative antagomir or miR-206 antagomir (dose, 40 mg/kg). Fish injected with PBS was taken as control. QRT-PCR analysis was used to detect relative expression, *U6* and *β-actin* expression level was detected as the internal control. Asterisk (*) indicates a significant difference (*P* < 0.05) in the same treatment before and after injection with Paired-samples t-test. Different lowercase letters show significant differences among treatments at each sampling point (*P* < 0.05, Duncan's multiple range tests).

**Figure 4:**
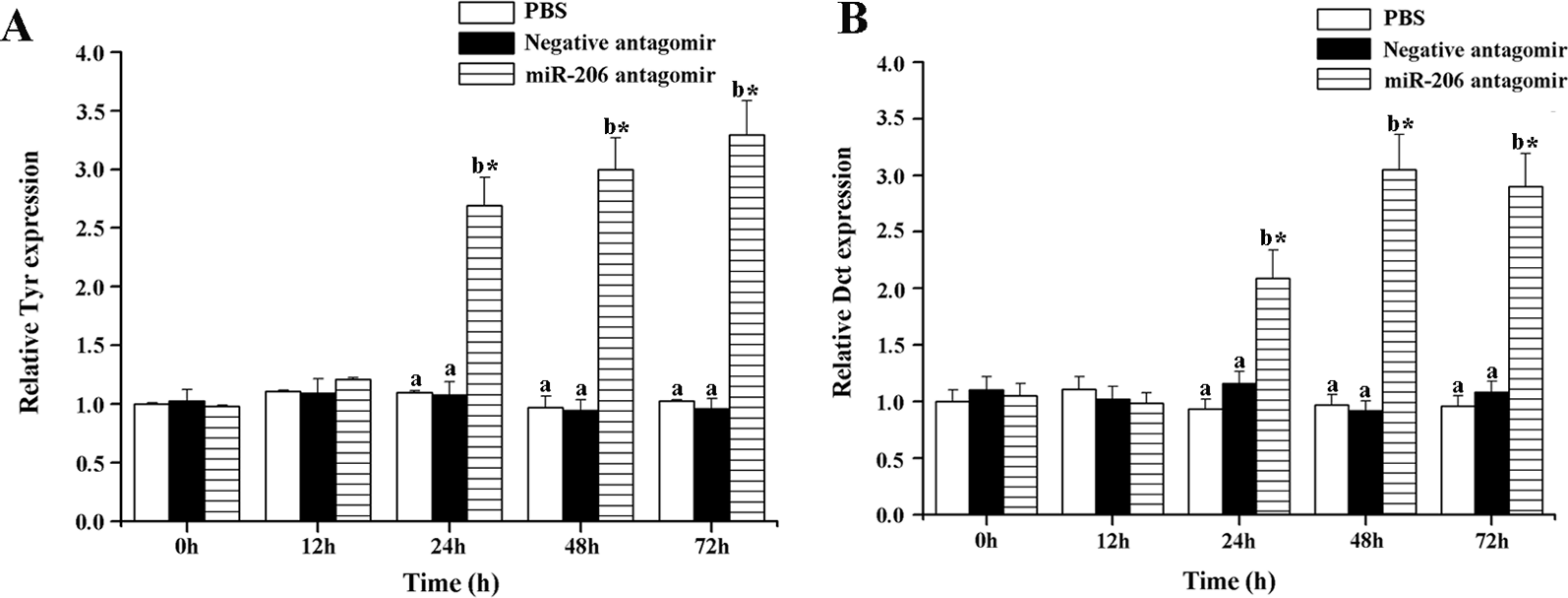
Effect of miR-206 silencing on the expression of *Tyr* **(A)** and *Dct* **(B)** genes. Asterisk (*) indicates a significant difference (*P* < 0.05) in the same treatment before and after injection with a paired-samples t-test. Different lowercase letters show significant differences among treatments at each sampling point (*P* < 0.05, Duncan's multiple range tests).

To further excavate the function of miR-206 in regulating pigmentation process *in vivo*, we then knocked down miR-206 with miRNA antagomir method through tail vein injection in the healthy koi carp. Firstly, we screened the optimal injection concentration, the interference efficiency and duration both were best in the 40 mg/kg group, based on the expression of miR-206 at different time points (Additional file 4, **Figure S3**). Furthermore, the silencing effect showed that miR-206 antagomir but not PBS nor negative antagomir treatment led to a significant decrease (*P* < 0.05) in endogenous miR-206 expression level after 12 h (**Figure 3A**). By contrast, *Mc1r* expression level was found to be significantly increased (*P* < 0.05) after 12 h in the miR-206 antagomir group, compared with the other groups (**Figure 3B**). Additionally, we also detected the *Mc1r* downstream genes, *Tyr* and *Dct*, were both markedly increased (*P* < 0.05) in the miR-206 antagomir group at 24 h post-injection compared with the other two groups (**Figures 4A, B**).

**Figure 5:**
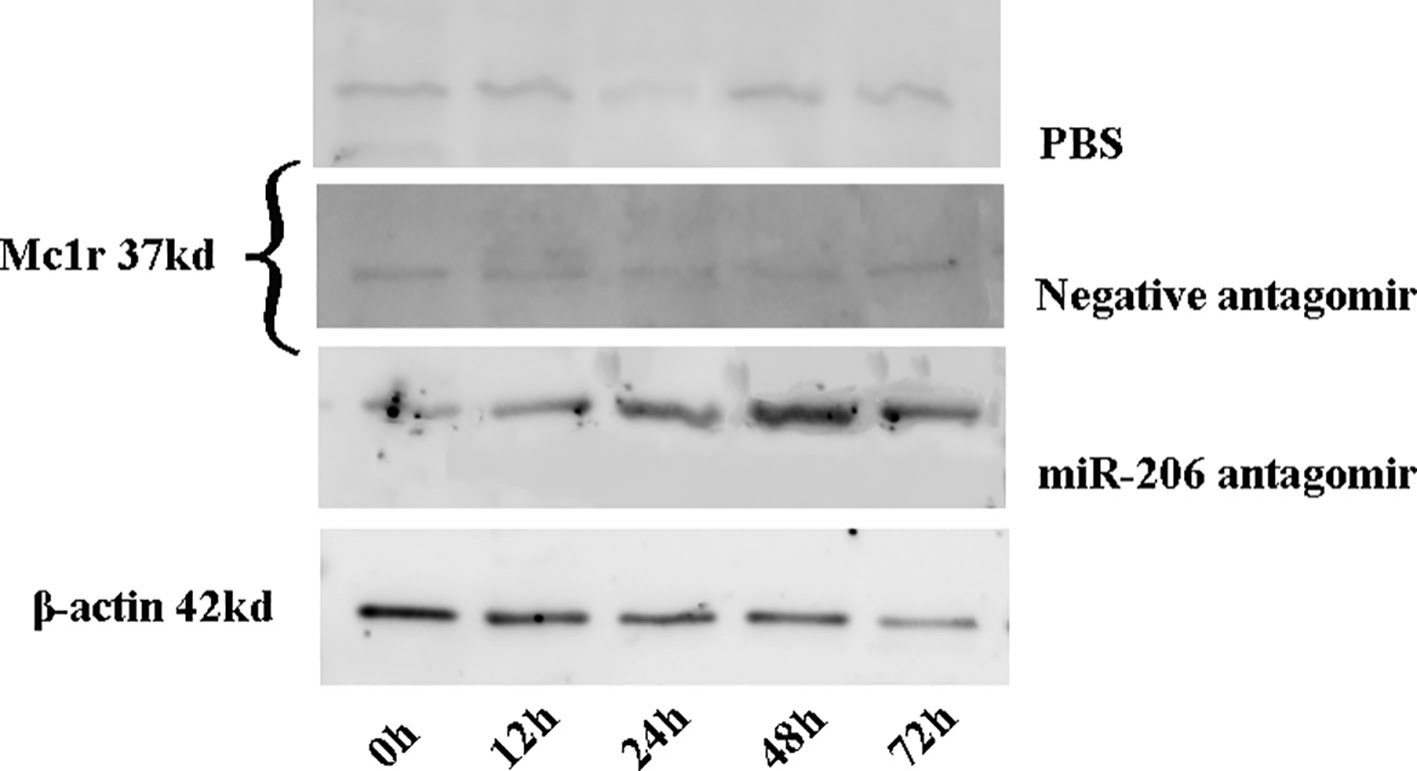
The Mc1r protein content of skin tissues in koi carp at each sampling time point was detected by western blot. β-actin was used as the internal control to quantify the change of Mc1r.

Meanwhile, the changes of Mc1r protein content at different time points were also detected in each treatment group. The results indicated a significant increase of Mc1r protein in the miR-206 antagomir group, compared with the PBS and negative antagomir groups (**Figure 5**). The inverse correlation between miRNA-206 and *Mc1r* expression at mRNA and protein level suggested that miR-206 directly regulates *Mc1r* expression *in vivo*.

### Interaction Between miR-206 and *Mc1r* in Melanocytes

**Figure 6:**
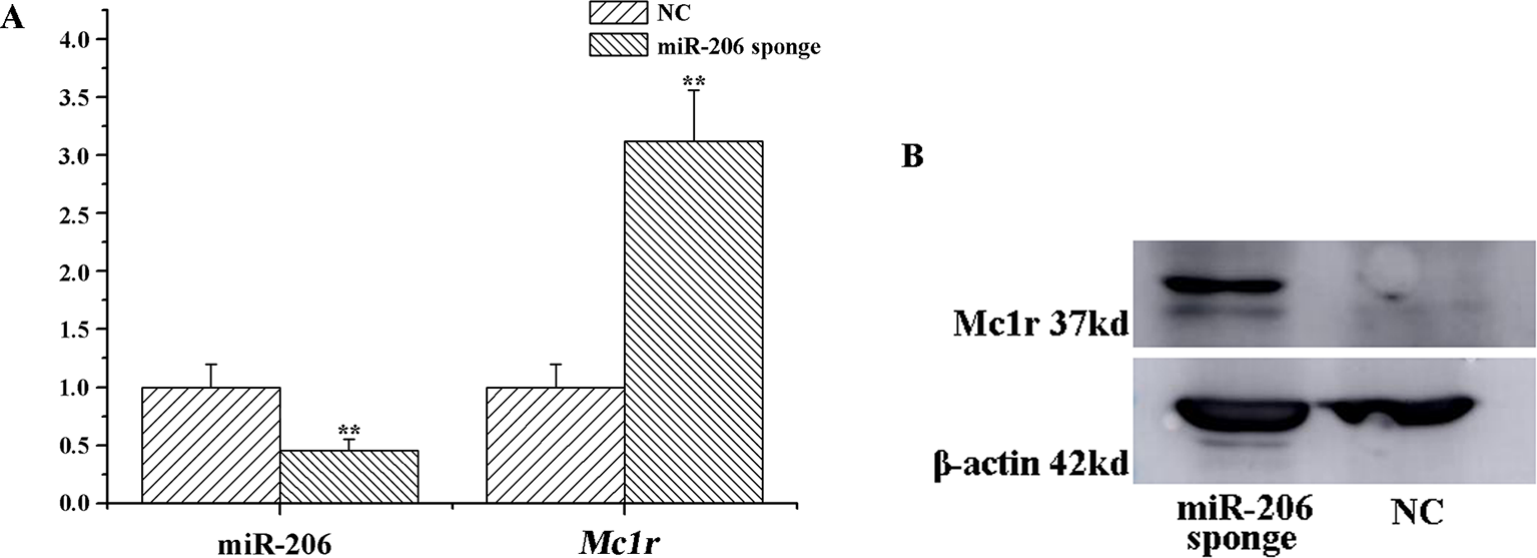
MiR-206 sponge promotes melanocytes proliferation. **(A)** Expression levels of miR-206 and *Mc1r* mRNA were determined by QRT-PCR after lentivirus transduction (**P < 0.01). **(B)** Western blot analysis of Mc1r protein content.

**Figure 7:**
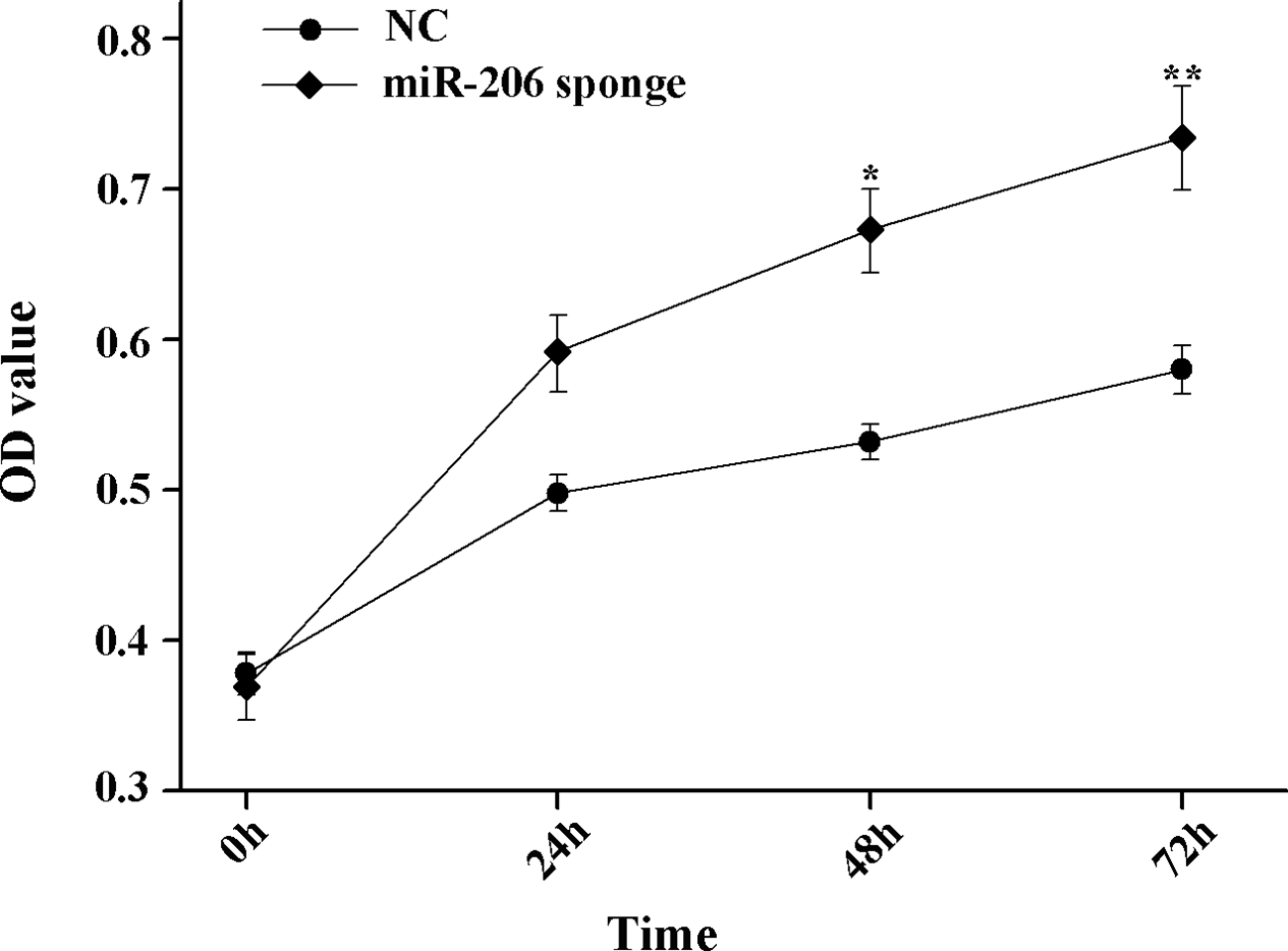
MiR-206 sponge promotes melanocytes proliferation. Cell proliferation was measured with OD value using a cell counting kit-8 (CCK-8) assay (*P < 0.05, **P < 0.01).

**Figure 8:**
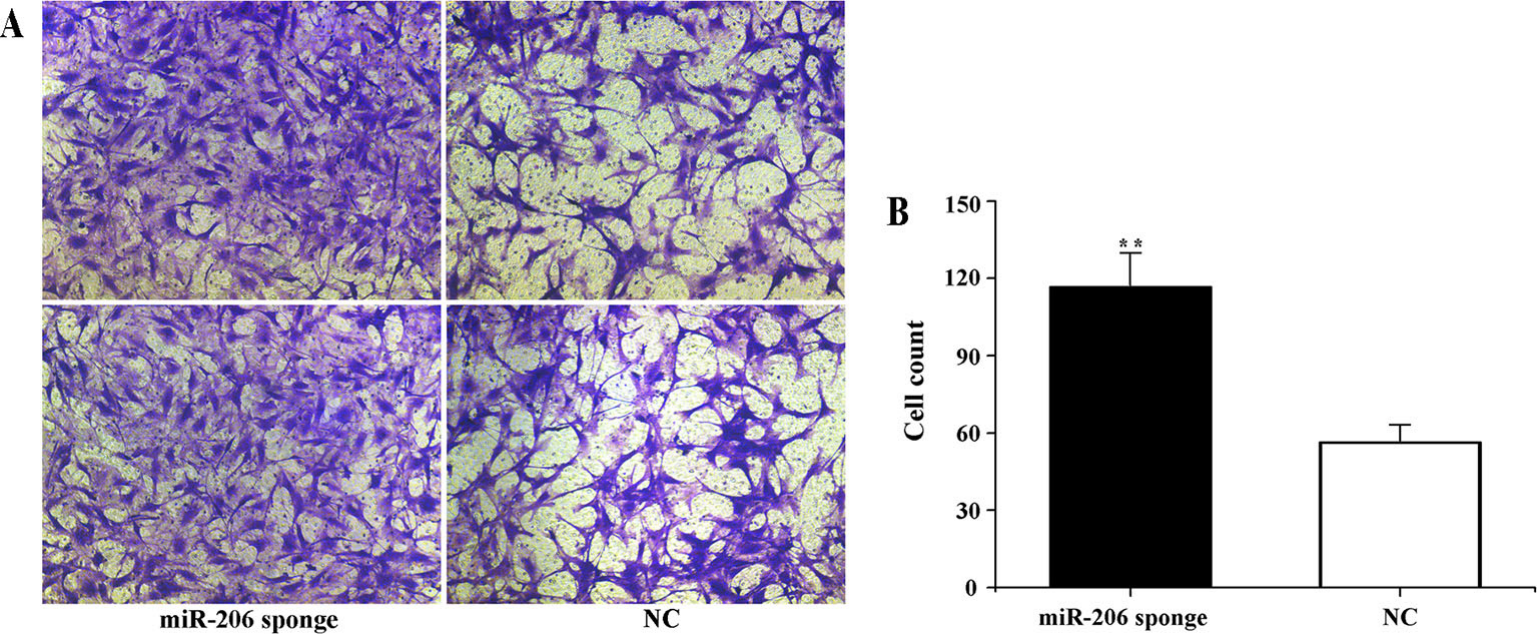
Effect of miR-206 sponge on koi carp melanocytes migration. **(A)** Representative images (100 ) of migrated cells of miR-206 sponge were effectively increased compared with a miR-NC group. The cell density of the miR-206 sponge group was higher than the NC group. **(B)** The count number of invading cells using cell counter (**P < 0.01).

To investigate the role of miR-206 in melanocytes progression, we constructed a miR-206 sponge plasmid and packaged it into the lentivirus vector. The koi carp melanocytes were transfected with either a miR-206 sponge knockdown vector or a scrambled-sequence lentivirus (negative control), both of which exhibited highly transfection efficiency (Additional file 4, **Figure S4**). Then, we detected the knockdown efficiency through qRT-PCR and western-blot method, the result showed that the expression level of miR-206 was significantly decreased in the miR-206 sponge group compared with the negative control (NC) (*P* < 0.01, **Figure 6A**). However, the silencing of miR-206 in melanocytes resulted in an increased expression level of *Mc1r* mRNA (*P* < 0.01, **Figure 6A**). On the other hand, the content of Mc1r protein in the miR-206 sponge transfected group was found increase when compared with the NC group (**Figure 6B**). Furthermore, we performed the cck-8 assay to detect whether miRNA-206 has an effect on melanocytes proliferation ability. The result revealed that when the miR-206 was knocked down, the melanocytes grew significantly faster than the miR-NC group (* *P* < 0.05, ** *P* < 0.01), the OD value of miR-206 sponge group was much higher after 24 h (**Figure 7**). Subsequently, we conducted a transwell assay to elucidate the effect of miR-206 on cell migration to further verify its function. The results demonstrated that the miR-206 sponge group was obviously accelerated melanocytes migration (**Figure 8A**), and its cell density was significantly higher (** *P* < 0.01) than miR-NC group after 48 h by checking the cells count number (**Figure 8B**).

## Discussion

Animals' coloration and their pattern is an important model to excavate the genetic mechanisms that determine phenotype (Joanna et al., 2010). In this study, we mainly focused on the miR-206 functional studies based on previous small RNA sequencing results (Luo et al., 2018). Firstly, we analyzed its temporal and spatial expression pattern to gain insight into the functions. The result showed that miR-206 displayed a dynamic expression pattern during embryonic development and was significantly up-regulated in the neurula stage. Interestingly, the research of our team regarding the expression levels of *Agouti*, *Mc1r*, *Mitf*, and *Tyr* mRNAs at early developmental stages of koi carp showed the same trend that peaked in gastrula and blastula periods, and then decreased (Yin et al., 2019). Given that the pigment stem cells are primarily derived from the neural crest during the gastrula stage (Parichy, 2006; Betancur et al., 2010), we guess both of them may play important roles in the formation of body colors in early embryonic development. Additionally, the result of expression level in different tissues indicated that miR-206 was expressed mainly in skin, muscle, and eyes. Meantime, some previous researches have also illustrated miR-206 was specifically expressed in skeletal and cardiac muscle, including mouse, rat, and zebrafish (Kim et al., 2006; Shan et al., 2009; Mishima et al., 2009). Furthermore, the *Mc1r* expression was found highest in black skin and eyes, followed by red, white skin, brain, muscle, and ovary in adult koi carp (Bar et al., 2013). At the same time, the *Mc1r* expression was also detected in all organs tested in platyfish and medaka but restricted to eyes, skin, brain, and testis in zebrafish, which possibly reflecting differences in the distribution of extracutaneous melanophores (Selz et al., 2007). Therefore, it would more crucially to excavate deeply into their molecular regulation mechanisms because different species have various expression patterns.

In animals, miRNAs target the mRNAs with partial sequence complementarily, the degree of pairing complementarily determines whether translation is subsequently repressed or the transcript is degraded (Karginov et al., 2007; Selbach et al., 2008). Here, we identified *Mc1r* 3'UTR exist the region that completely matched an 9-nt “seed sequence”, i.e. 8-16 site, at the 3' end of the miR-206. Then, we conducted a dual-luciferase reporter assay system to confirm the binding site at the cellular level. The relative activity of the luciferase reporter linked to target *Mc1r* was significantly decreased when miR-206 was over-expressed, while the luciferase activity has no obvious variation in a mutation reporter vector of the target site. Thus we further confirmed that there were specific recognition elements between miR-206 and *Mc1r*.

Antagomirs are cholesterol-conjugated single-stranded RNA molecules (21-23 nt) that complementary to the specific miRNA region with either mispairing at the cleavage of Ago2 or some varieties of base modification to inhibit Ago2 cleavages (Krutzfeldt et al., 2005). Some previous studies have revealed that the antagomirs could block the endogenous miRNA expression in cell lines, zebrafish, and tilapia (Morton et al., 2008; Selvamani et al., 2012; Qiang et al., 2017). Therefore, we also conducted this method to verify whether it would impede endogenous miR-206 expression and effect on the target *Mc1r* gene. We found miR-206 expression level was significantly decreased, while the *Mc1r* gene was marked up-regulation and its protein content also increased, suggesting there exists a targeted regulation relationship between them. *Mc1r* is one member of the G-protein-coupled receptor family with a characteristic tissue distribution pattern and a unique biological function (Jose et al., 2005; Cone, 2006). When MC1R interacts with the α-melanocyte stimulating hormone (*α-MSH*) ligand, the protein would activate adenylate cyclase to stimulate transcription of the *mitf* gene, including those encoding key enzymes like *Tyr*, *Tyrp1*, *Dct*, etc. which involved in the melanin synthesis (Yamaguchi et al., 2007). Previous study illustrated that mutation in *Mc1r* gene would have large effect on the vertebrate coloration, which are important in the origin of new species or local adaptation within species (Beaumont et al., 2007). These shreds of evidence suggest that *Mc1r* expression is tightly correlated with the development of pigment cells. Therefore, we further detected the downstream genes (*Tyr* and *Dct*) to verify whether the expression level would change due to the silence of miR-206 *in vivo*. The results finally illustrated that their expression levels were both markedly up-regulated compared with control groups, which revealed miR-206 might play an important role in the melanogenesis pathway.

To date, a few miRNAs have been characterized to act as crucial roles in governing the regulation of melanin. For example, miR-434-5p was identified to inhibit the *Tyr* mRNA expression level, which led to mouse skin turn white (Wu et al., 2008). Wang et al. transfected the miR-21a-5p into mouse melanocytes and demonstrated its function on melanogenesis through targeting *Sox5* mRNA, which inhibits melanogenesis in mouse melanocytes (Wang et al., 2016). Over-expression or down-regulation of miR-145 in mouse melan-α cells were led to reduce or increase expression levels of *Sox9*, *Mitf*, *Tyr*, *Tyrp1*, *Myo5a*, and *Fscn1*, suggesting miR-145 played an important role in melanogenesis (Peter et al., 2013). In our study, we finally constructed a miR-206 sponge lentivirus for transfection into the koi carp melanocytes, then uncovered whether miR-206 silencing could increase cell migration and proliferation. The results showed that there were no changes in cell morphology, color, and size; only the number of cells was changed and the melanocytes migration capacity of miR-206 sponge group significantly higher than the miR-NC group after 48 h. Indeed, other studies have also found that *Mc1r* regulates such migration, and also influenced by its ligands. For example, Heesung et al. found the expression level of *Mc1r* was closely related to the migration extent of mouse melanoma cells, which suggests *Mc1r* acts as a functional role in controlling this migration. And over-expression of *Mc1r* would enhance melanoma cell migration, whereas the opposite was true when knocked down the *Mc1r* using small inhibitory RNAs (Chung et al., 2012). More than 80% of human metastatic melanoma cell lines were found expression of *Mc1r*, and there have high levels in primary and metastatic melanoma tissue samples through immunohistochemistry analyses (Salazar-Onfray et al., 2002). The melanoma cell migration was also reduced by the *α-Msh* (Zhu et al., 2004), and this inhibition appear to be associated with the development of an anti-inflammatory response (Eves et al., 2003). Therefore, we can see *Mc1r* appears to play a receptor potentiating melanoma cell migration. Moreover, we demonstrated miR-206 negatively regulated the *Mc1r* mRNA expression level and protein content, suggesting that miR-206 might play a crucial role in governing the regulation of the melanogenesis process in koi carp.

## Conclusions

Taken together, we found a new miRNA regulatory mechanism for skin pigmentation in koi carp. We detected that miR-206 was highly enriched in the skin, muscle, eyes, and neurula stage. Treatment with miR-206 antagomir resulted in a significantly decrease of endogenous miR-206 level, and an obvious increase of *Mc1r* mRNA expression, Mc1r protein, and its downstream genes including *Tyr*, *Dct* that involved in the melanogenesis pathway. Then, we demonstrated the miR-206 sponge lentivirus vector would increase the *Mc1r* mRNA expression level and protein content, which appears to act as a receptor potentiating melanocytes migration and proliferation. Overall, the present work provides the evidence that miR-206 play a regulatory role in the skin color pigmentation through targeting the *Mc1r* gene and shows its promise as a potential molecular target to assist the selection of koi carp with better skin color patterns.

## Materials and Methods

### Sample

Koi carps were obtained from the Qiting Pilot Research Station (Yixing, Jiangsu, China), affiliated to the Freshwater Fisheries Research Center (FFRC). They were kept in 256-liter tanks of a water circulation system under a 12-h light/dark photoperiod at 24 ± 1°C and fed twice daily with compound feed.

Fish were tranquilized in 10~15 mg/L MS-222 buffered to pH 7.0~7.5, then skin tissues (black, red, and white) and the other nine tissue samples (gonad, gill, liver, muscle, blood, heart, eye, brain, and kidney) were collected. The embryonic developmental stage samples were collected at the aquaculture base during the reproduction periods, and their classify criteria referred to the Kimmel et al.'s article (Kimmel et al., 1995). All of samples were immediately snap-frozen in liquid nitrogen, then stored at −80°C until use.

### Cell Culture

The koi carp melanocytes and HEK293T cell lines were purchased from the Umibio Co., Ltd. Shanghai, China. Then the melanocytes were cultured in RPMI-1640 and HEK293T cells were grown in DMEM (Gibco, Grand Island, USA). All mediums were supplemented with 10% fetal bovine serum (FBS) (Invitrogen, Carlsbad, CA, USA) and 1% penicillin/streptomycin (P/S, Gibco). The cells were incubated at 37°C under 5% CO_2_.

### Luciferase Reporter Assay

The 3'-UTR of *Mc1r* was synthesized referred to the sequence of common carp in GenBank (JX989223.1) and individually cloned into pSE3575 vector (Sunbio Medical Biotech Co., Ltd. Shanghai, China) by directional cloning. The mutant *Mc1r* 3'-UTR reporters were constructed by mutating the seed region (ATTCCTTCT to ATCTTCCTT) of the predicted miR-206 site. Then, 25 ng of the luciferase reporter vector containing 50 nM miR-206 mimic, miR-206 wild-type (wt), or the 3'-UTR mutant was co-transfected with 5 ng *Renilla* luciferase control vector (pRL-TK, Promega, Madison, WI, USA) using lipofectamine 2000 (Invitrogen, Carlsbad, CA, USA) in 24-well plates. Luciferase assays were carried out at 48 h post-transfection through the Dual-Luciferase Reporter Assay System (Promega, Madison, WI, USA) according to the manufacturer's protocols. A liquid scintillation counter was applied to detect the luciferase activity, the Firefly Luciferase activity was normalized to the *Renilla* luciferase activity.

### Silencing miR-206 *In Vivo*

The antagomir used in our study was single-stranded RNA, which consist of 21~23nt modified as follows: antagomir-206, CsCsACACACACUUCCUUACAUUs CsCsAs-Chol-3' and negative antagomir, CsAsCGGUUCCAGGCACUGUsGsUsAs-Chol-3'. The “s” represents a phosphorothioate linkage and “Chol” represents cholesterol linked through a hydroxyproline linkage. All nucleotides were 2'-OMe-modified.

The healthy koi carps (each about 10 g) were injected into the tail vein with miR-206 antagomir, negative antagomir, and phosphate-buffered saline (PBS) (control), respectively. Each treatment had three replicates. At 0, 12, 24, 48, 72 h after treatment, skin tissues were sampled, immediately snap-frozen in liquid nitrogen, and stored at −80°C.

### Plasmid Construction and Lentivirus Preparation

The full length of miRNA-206 sponge sequence was chemically synthesized (5'-CCACACACTTCCTTACATTCCActcgagCCACACACTTCCTTACATTCCAggatccCCACACACTTCCTTACATTCCAgggccc CCACACACTTCCTTACATTCCAggatccCCACACACTTCCTTACATTCCActcgagCCACACACTTCCTTACATTCCA-3') based on optimizations previously described (Kluiver et al., 2012a; Kluiver et al., 2012b). Then, the sequence was inserted into the FV115 lentiviral vector (Additional file 1, **Figure S1**), which purchased from the Umibio Co., Ltd. China within the EcoRI and XbaI restriction sites to construct a lentivirus encoding miR-206. The aforementioned lenti-miR-206 plasmid, pHelper 1.0, and pHelper 2.0 were co-transfected into 293T cells, and the lentiviral-particle-enriched supernatant was obtained at 48 h later (Additional file 1, **Figure S2**). Furthermore, the melanocytes were seeded in a six-well plates with a density of 5 × 10^4^ cells/well, and infected with lenti-miR-206 when the cells reached 60~70% confluence. After 48 h of infection, a complete medium containing 3 µg/ml puromycin was replaced for culture and an empty lentiviral vector was also used as a negative control. Finally, the efficiency of infection was evaluated by observing the GFP expression and frozen in liquid nitrogen when the cell density grew to 90%.

### Cck8 and Transwell Assay

Cell proliferation capacity was analyzed through Cell Counting Kit-8 (CCK, Dojindo Molecular Technologies Inc., Japan). Melanocytes were digested with 0.25% trypsin, then seeded in 96-well plates with 2 × 10^3^ cells/well, treated with cck8 solution (10 µl/well) for 24, 48, or 72 h. The cells were continuously incubated for 4 h at 37°C under 5% CO_2_. Finally, the absorbance was measured at 450 nm by a microplate reader (Molecular Devices, CA, USA). For transwell migration assays, 1 × 10^5^ cells (lentivirus infected or negative control) were plated in the top chamber onto the noncoated membrane (24-well insert; pore size, 8 µm; Corning Corporation, MA, USA). Then, a total of 600 µl medium containing 30% FBS was added to the lower chamber. And 24 h later of incubation, cells on the lower surface of the membrane were fixed in 90% methanol for 10 min and stained with 0.1% crystal violet, counted in three random shots by microscopy, and photographed (Olympus). The numbers of cells were counted at 100 magnification using cell counter. All experiments were performed in triplicate.

### RNA Isolation and Quantitative PCR

MiRNAs were extracted through a miRNeasy Kit (Takara, China) and were reverse transcribed using the Mir-X™ miRNA First-strand Synthesis Kit (Takara) according to the manufacturer's protocol. QRT-PCR assay was performed on a CFX-96 Real-time PCR System (Bio-Rad, CA, USA) in 25 µl reactions including 12.5 µl SYBR Advantage Premix (2×) reagent (Takara), 0.5 µl miRNA-specific forward primer (10 µM), 0.5 µl miScript universal primer (10 µM), and 2 µl PCR template (cDNA). Then, the amplification was conducted with an initial activation at 95°C for 10 s, followed by 40 cycles of 95°C for 5 s and 60°C for 20 s, with a final cycle from 95°C to 65°C. *U6* snRNA was used as an internal reference.

Total RNAs were also isolated using Trizol reagent (Invitrogen, Carlsbad, CA, USA) according to the manufacturer's protocol. Then measured by a Nanodrop 1000 spectrophotometer (Thermo Scientific, DE, USA) with an OD_260_/OD_280_ ratio between 1.8~2.0 used for cDNA synthesis (Takara). And qPCR assay was also performed on a CFX-96 Real-time PCR System according to the manufacture's protocol (Takara). All primers (Additional file 2, **Table S1**) used in our study were designed using Primer Premier 5 and synthesized by Sangon Biotech., China. Relative mRNA or miRNA expression level was calculated using the comparative Ct method, which is also referred to as the 2^–ΔΔCt^ method (Chen et al., 2005; Schmittgen and Livak, 2008). All reactions were conducted in triplicate.

### Western Blot

Protein samples were obtained from the skin tissues and melanocytes according to the Protein Extraction Kit (Sangon Biotech., China). Protein content was quantified using the Bio-Rad protein assay. Then, samples were diluted, heated for denaturation, and subjected to SDS-polyacrylamide gel electrophoresis (SDS-PAGE). After that, the proteins were transferred to a polyvinylidene difluoride (PVDF) membrane (Millipore, MA, USA) and blocked with Tris-buffered saline containing 0.05% Tween-20 (TBST) and 5% non-fat dry milk for 1 h. Then, 1:1,000 dilutions of rabbit polyclonal primary antibody anti-*mc1r*-C (HuaAn Biotechnology Co., Ltd., China) or anti-β-actin (loading control) were incubated at 4°C overnight. The following day, the membrane was rinsed in TBST three times and incubated with a fluorescent secondary anti-rabbit antibody (1:5,000) for 1 h at 37°C. After washing three times with Tris-buffered saline at 5 min per wash, the proteins were detected using ECL chemiluminescence (Beyotime, China) by image software (Bio-Rad Laboratories, PA, USA).

### Statistical Analysis

All results were expressed as means ± standard deviation. All data were analyzed by SPSS software version 21.0. (SPSS Inc., IL, USA). The sampling points of different treatments were analyzed by one-way ANOVA and a *post hoc* Duncan's multiple range tests. Different sampling points with each treatment were statistically analyzed by the t-test. *P* < 0.05 was deemed to be significant.

## Data Availability Statement

The raw data supporting the conclusions of this article will be made available by the authors, without undue reservation, to any qualified researcher.

## Ethics Statement

The animal study was reviewed and approved by Animal Care and Use committee of the Centre for Applied Aquatic Genomics at the Chinese Academy of Fishery Sciences (CAFS) (BC 2013863, 9/2013).

## Author Contributions

ZD designed and supervised the study. ML performed experiments and analyzed the data. ZD, LW, and WZ analyzed the data and prepared tables and figures. JF and HY performed qRT-PCR experiments. ML wrote the manuscript. All authors read, reviewed, and approved the manuscript for submission.

## Funding

This study was supported by the Central Public Interest Scientific Institution Basal Research Fund, CAFS (NO.2019ZY22), the “333 Project” Training Fund Project of Jiangsu Province (BRA2017083) and Jiangsu Provincial Postdoctoral Research Program in 2018 (2018K208C). The funders had no role in study design, data collection, analysis, interpretation of data, or manuscript preparation.

## Conflict of Interest

The authors declare that the research was conducted in the absence of any commercial or financial relationships that could be construed as a potential conflict of interest.

## References

[B1] Altschmied J. Delfgaauw J. Wilde B. Duschl J. Bouneau L. Volff J. N. (2002). Subfunctionalization of duplicate mitf genes associated with differential degeneration of alternative exons in fish. Genetics 161 (1), 259–267. 12019239 10.1093/genetics/161.1.259 PMC1462118

[B2] Ambros V. (2004). The functions of animal microRNAs. Nature 431, 350–355. 10.1038/nature02871 15372042

[B3] Bandyopadhyay S. Mitra R. (2009). TargetMiner: microRNA target prediction with systematic identification of tissue-specific negative examples. Bioinformatics 25 (20), 2625–2631. 10.1093/bioinformatics/btp503 19692556

[B4] Bar I. Kaddar E. Velan A. David L. (2013). Melanocortin receptor 1 and black pigmentation in the Japanese ornamental carp (*Cyprinus carpio* var. koi). Front. Genet. 4, 6. 10.3389/fgene.2013.00006 23355846 PMC3554881

[B5] Beaumont K. A. Shekar S. L. Newton R. A. James M. R. Stow J. L. Duffy D. L. (2007). Receptor function, dominant negative activity and phenotype correlations for Mc1r variant alleles. Hum. Mol. Genet. 16, 2249–2260. 10.1093/hmg/ddm177 17616515

[B6] Betancur P. Bronner F. M. Sauka S. T. (2010). Assembling neural crest regulatory circuits into a gene regulatory network. Annu. Rev. Cell Dev. Bi. 26 (1), 581–603. 10.1146/annurev.cellbio.042308.113245 PMC4040144 19575671

[B7] Braasch I. Schartl M. Volff J. N. (2007). Evolution of pigment synthesis pathways by gene and genome duplication in fish. BMC Evol. Biol. 7, 74. 10.1186/1471-2148-7-74 17498288 PMC1890551

[B8] Braasch I. Liedtke D. Volff J. N. Schartl M. (2009). Pigmentary function and evolution of *tyrp1* gene duplicates in fish. Pigm. Cell Melanoma Res. 22, 839–850. 10.1111/j.1755-148X.2009.00614.x 19659755

[B9] Ceol C. J. Houvras Y. White R. M. Zon L. I. (2008). Melanoma biology and the promise of zebrafish. Zebrafish> 5 (4), 247–256. 10.1089/zeb.2008.0544 19133823 PMC2784934

[B10] Chen C. Ridzon D. A. Broomer A. J. Zhou Z. Lee D. H. Nguyen J. T. (2005). Real-time quantification of microRNAs by stem-loop RT-PCR. Nucleic Acids Res. 33, e179. 10.1093/nar/gni178 16314309 PMC1292995

[B11] Chistiakov D. A. Voronova N. V. (2009). Genetic evolution and diversity of common carp *Cyprinus carpio* L. Cent. Eur. J. Biol. 4 (3), 304–312. 10.2478/s11535-009-0024-2

[B12] Chung H. Lee J. H. Jeong D. Han I. O. Oh E. S. (2012). Melanocortin 1 receptor regulates melanoma cell migration by controlling syndecan-2 expression. J. Biol. Chem. 287 (23), 19326–19335. 10.1074/jbc.M111.334730 22493442 PMC3365964

[B13] Cone R. D. (2006). Studies on the physiological functions of the melanocortin system. Endocr. Rev. 27, 736–749. 10.1210/er.2006-0034 17077189

[B14] Damme P. V. Appelbaum S. Hecht T. (2010). Sibling cannibalism in koi carp, *Cyprinus carpio* L. larvae and juveniles reared under controlled conditions. J. Fish Biol. 34 (6), 855–863. 10.1111/j.1095-8649.1989.tb03369.x

[B15] Dong C. Wang H. Xue L. Dong Y. Yang L. Fan R. (2012). Coat color determination by miR-137 mediated down-regulation of microphthalmia-associated transcription factor in a mouse model. RNA 18, 1679–1686. 10.1261/rna.033977.112 22847819 PMC3425782

[B16] Eves P. Haycock J. Layton C. Wagner M. Kemp H. Szabo M. (2003). Anti-inflammatory and anti-invasive effects of α-melanocyte-stimulating hormone in human melanoma cells. Br. J. Cancer. 89, 2004–2015. 10.1038/sj.bjc.6601349 14612916 PMC2394449

[B17] Grimson A. Farh K. K. H. Johnston W. K. Garrett-Engele P. Lim L. P. Bartel D. P. (2007). MicroRNA targeting specificity in mammals: determinants beyond seed pairing. Mol. Cell. 27 (1), 91–105. 10.1016/j.molcel.2007.06.017 17612493 PMC3800283

[B18] Hoekstra H. E. (2006). Genetics, development and evolution of adaptive pigmentation in vertebrates. Heredity 97 (3), 222–234. 10.1038/sj.hdy.6800861 16823403

[B19] Hwang H. W. Mendell J. T. (2006). MicroRNAs in cell proliferation, cell death, and tumor-genesis. Br. J. Cancer. 94, 776–780. 10.1038/sj.bjc.6603023 16495913 PMC2361377

[B20] Jha P. Barat S. (2006). Nayak C R. A comparison of growth, survival rate and number of marketable koi carp produced under different management regimes in earthen ponds and concrete tanks. Aquacult. Int. 14 (6), 615–626. 10.1007/s10499-006-9059-9

[B21] Joanna K. H. Uy J. A. C. Hauber M. E. Hoekstra H. E. Safran R. J. (2010). Vertebrate pigmentation: from underlying genes to adaptive function. Trends Genet. 26 (5), 231–239. 10.1016/j.tig.2010.02.002 20381892

[B22] Jose C. Sánchez-Laorden B. L. Jiménez-Cervantes C. (2005). Melanocortin-1receptor structure and functional regulation. Pigm. Cell Res. 18(6), 393–410. 10.1111/j.1600-0749.2005.00278.x 16280005

[B23] Karginov F. V. Conaco C. Xuan Z. Schmidt B. H. Parker J. S. Mandel G. (2007). A biochemical approach to identifying microRNA targets. P. Natl. Acad. Sci. USA. 104 (49), 19291–19296. 10.1073/pnas.0709971104 PMC2148283 18042700

[B24] Kelsh R. N. (2004). Genetics and evolution of pigment patterns in fish. Pigm. Cell Res. 17, 326–336. 10.1111/j.1600-0749.2004.00174.x 15250934

[B25] Kennell J. A. Cadigan K. M. Shakhmantsir I. Waldron E. J. (2012). The microRNA miR-8 is a positive regulator of pigmentation and eclosion in *Drosophila* . Dev. Dyn. 241, 161–168. 10.1002/dvdy.23705 22174085 PMC3243916

[B26] Kim H. K. Lee Y. S. Sivaprasad U. Malhotra A. Dutta A. (2006). Muscle specific microRNA miR-206 promotes muscle differentiation. J. Cell Biol. 174, 677–687. 10.1083/jcb.200603008 16923828 PMC2064311

[B27] Kimmel C. B. Ballard W. W. Kimmel S. R. Ullmann B. Schilling T. F. (1995). Stages of embryonic development of the zebrafish. Dev. Dyn. 203 (3), 253–310. 10.1002/aja.1002030302 8589427

[B28] Kluiver J. Gibcus J. H. Hettinga C. Adema A. Richter M. K. Halsema N. (2012a). Rapid generation of microRNA sponges for microRNA inhibition. PloS One 7 (1), e29275. 10.1371/journal.pone.0029275 22238599 PMC3253070

[B29] Kluiver J. Slezak-Prochazka I. Smigielska-Czepiel K. Halsema N. Kroesen B. J. Van den Berg A. (2012b). Generation of miRNA sponge constructs. Methods 58 (2), 113–117. 10.1016/j.ymeth.2012.07.019 22836127

[B30] Krol J. Loedige I. Filipowicz W. (2010). The widespread regulation of microRNAs biogenesis, function and decay. Nat. Rev. Genet. 11, 597–610. 10.1038/nrg2843 20661255

[B31] Krutzfeldt J. Rajewsky N. Braich R. (2005). et al. Silencing of microRNAs *in vivo* with ‘antagomirs'. Nature 438, 685–689. 10.1038/nature04303 16258535

[B32] Lin S. Pan Y. Luo L. Luo L. (2011). Effects of dietary β-1,3-glucan, chitosan or raffinose on the growth, innate immunity and resistance of koi (*Cyprinus carpio* L.). Fish Shellfish Immun. 31 (6), 788–794. 10.1016/j.fsi.2011.07.013 21784160

[B33] Liu J. H. Wen S. Luo C. Zhang Y. Q. Tao M. Wang D. W. (2015). Involvement of the *Mitfa* gene in the development of pigment cell in Japanese ornamental (koi) carp (*Cyprinus carpio* L.). Genet. Mol. Res. 14 (1), 2775–2784. 10.4238/2015.March.31.7 25867426

[B34] Luo M. Wang L. Zhu W. Fu J. Song F. Fang M. (2018). Identification and characterization of skin color microRNAs in koi carp (*Cyprinus carpio* L.) by Illumina sequencing. BMC Genomics 19, 779. 10.1186/s12864-018-5189-5 30373521 PMC6206873

[B35] Luo M. Wang L. Yin H. Zhu W. Fu J. Dong Z. (2019). Integrated analysis of long non-coding RNA and mRNA expression in different colored skin of koi carp. BMC Genomics 20, 515. 10.1186/s12864-019-5894-8 31226932 PMC6588874

[B36] Manas K. M. Bora D. Nandeesha T. L. Sahoo S. Adarsh B. K. Kumar S. (2017). Effect of dietary natural carotenoid sources on colour enhancement of Koi carp, Cyprinus carpio L. Int. J. Fish. Aquat. Stud. 5 (4) 340–345

[B37] McGraw K. J. Safran R. J. Wakamatsu K. (2005). How feather color reflects its melanin content. Funct. Ecol. 19, 816–821. 10.1111/j.1365-2435.2005.01032.x

[B38] Mishima Y. Abreu-Goodger C. Staton A. A. Stahlhut C. Shou C. Cheng C. (2009). Zebrafish miR-1 and miR-133 shape muscle gene expression and regulate sarcomeric actin organization. Genes Dev. 23, 619–632. 10.1101/gad.1760209 19240126 PMC2658521

[B39] Morton S. U. Scherz P. J. Cordes K. R. Ivey K. N. Stainier D. Y. Srivastava D (2008). microRNA-138 modulates cardiac patterning during embryonic development. P. Natl. Acad. Sci. U. S. A. 105, 17830–17835. 10.1073/pnas.0804673105 PMC2582580 19004786

[B40] Parichy D. M. (2006). Evolution of *danio* pigment pattern development. Heredity 97 (3), 200–210. 10.1038/sj.hdy.6800867 16835593

[B41] Peter D. Mestdagh P. Van Peer G. Vandesompele J. Goossens K. Peelman L. J. (2013). Identification of miR-145 as a key regulator of the pigmentary process. J. Invest. Dermatol. 133, 201–209. 10.1038/jid.2012.266 22895360

[B42] Protas M. E. Patel N. H. (2008). Evolution of coloration patterns. Annu. Rev. Cell Dev. Biol. 24, 425–446. 10.1146/annurev.cellbio.24.110707.175302 18593352

[B43] Qiang J. Tao Y. F. He J. Sun Y. L. Xu P. (2017). miR-29a modulates SCD expression and is regulated in response to a saturated fatty acids diet in juvenile GIFT (*Oreochromis niloticus*). J. Exp. Bio. 220 (8), 1481–1489. 10.1242/jeb.151506 28167804 PMC5413068

[B44] Salazar-Onfray F. Lopez M. Lundqvist A. Aguirre A. Escobar A. Serrano A. (2002). Tissue distribution and differential expression of melanocortin 1 receptor, a malignant melanoma marker. Br. J. Cancer. 87, 414–422. 10.1038/sj.bjc.6600441 12177778 PMC2376124

[B45] Schmittgen T. D. Livak K. J. (2008). Analyzing real-time PCR data by the comparative CT method. Nat. Protoc. 3, 1101–1108. 10.1038/nprot.2008.73 18546601

[B46] Selbach M. Schwanhäusser B. Thierfelder N. Fang Z. Khanin R. Rajewsky N. (2008). Widespread changes in protein synthesis induced by microRNAs. Nature 455 (7209), 58–63. 10.1038/nature07228 18668040

[B47] Selvamani A. Sathyan P. Miranda R. C. (2012). et al. An antagomir to microRNA Let7f promotes neuroprotection in an ischemic stroke model. PloS One 7, e32662. 10.1371/journal.pone.0032662 22393433 PMC3290559

[B48] Selz Y. Braasch I. Hoffmann C. Schmidt C. Schultheis C. Schartl M. (2007). Evolution of melanocortin receptors in teleost fish: the melanocortin type 1 receptor. Gene 401 (1-2), 114–122. 10.1016/j.gene.2007.07.005 17707598

[B49] Shan Z. X. Lin Q. X. Fu Y. H. Deng C. Y. Zhou Z. L. Zhu J. N. (2009). Upregulated expression of miR-1/miR-206 in a rat model of myocardial infarction. Biochem. Biophys. Res. Commun. 381, 597–601. 10.1016/j.bbrc.2009.02.097 19245789

[B50] Wang C. H. Li S. F. (2004). Phylogenetic relationships of ornamental (koi) carp, Oujiang color carp and Long-fin carp revealed by mitochondrial DNA COII gene sequences and RAPD analysis. Aquaculture 231 (1-4), 83–91. 10.1016/j.aquaculture.2003.10.040

[B51] Wang P. Zhao Y. Fan R. Chen T. Dong C. (2016). MicroRNA-21a-5p functions on the regulation of melanogenesis by targeting sox5 in mouse skin melanocytes. Int. J. Mol. Sci. 17, 959. 10.3390/ijms17070959 PMC4964364 27347933

[B52] Wang F. Jia Y. Wang P. Yang Q. Du Q. Chang Z. (2017). Identification and profiling of *Cyprinus carpio* microRNA during ovary differentiation by deep sequencing. BMC Genomics 18 (1), 333. 10.1186/s12864-017-3701-y 28454515 PMC5410099

[B53] Wu D. T. Chen J. S. Chang D. C. Lin S. L. (2008). miR-434-5p mediates skin whitening and lightening. Clin. Cosmet Invest. Dermatol. 1, 19–35. 10.2147/CCID.S4181 PMC3048595 21437136

[B54] Xu P. Zhang X. Wang X. Li J. Liu G. Kuang Y. (2014). Genome sequence and genetic diversity of the common carp, *Cyprinus carpio* . Nat. Genet. 46 (11), 1212–1219. 10.1038/ng.3098 25240282

[B55] Yamaguchi Y. Brenner M. Hearing V. J. (2007). The regulation of skin pigmentation. J. Biol. Chem. 282, 27557–27561. 10.1074/jbc.R700026200 17635904

[B56] Yan B. Liu B. Zhu C. D. Li K. L. Yue L. J. Zhao J. L. (2013). MicroRNAs regulation of skin pigmentation in fish. J. Cell Sci. 126, 3401–3408. 10.1242/jcs.125831 23729736

[B57] Yi R. Poy M. N. Stoffel M. Fuchs E. (2008). A skin microRNA promotes differentiation by repressing ‘stemness'. Nature 452, 225–229. 10.1038/nature06642 18311128 PMC4346711

[B58] Yin H. R. Luo M. K. Wang L. M. Dong Z. J. Zhu W. B. Fu J. J. (2019). Changes of pigment-related enzyme activity and gene expression at early developmental stage of koi carp. South China Fish. Sci. 15 (5), 109–117. 10.12131/20190023

[B59] Zhu N. Lalla R. Eves P. Brown T. L. H. King A. Kemp E. H. (2004). Melanoma cell migration is upregulated by tumour necrosis factor-α and suppressed by α-melanocyte-stimulating hormone. Br. J. Cancer. 90, 1457–1463. 10.1038/sj.bjc.6601698 15054471 PMC2409669

