# Supplementary material for: MicroRNA-206 Regulation of Skin Pigmentation in Koi Carp (Cyprinus carpio L.)
Source: Front Genet. 2020 Feb 12;11:47. doi: 10.3389/fgene.2020.00047 (PMC7029398; doi:10.3389/fgene.2020.00047)
Supplement: Supplementary file 1 [file Presentation_1.pdf]

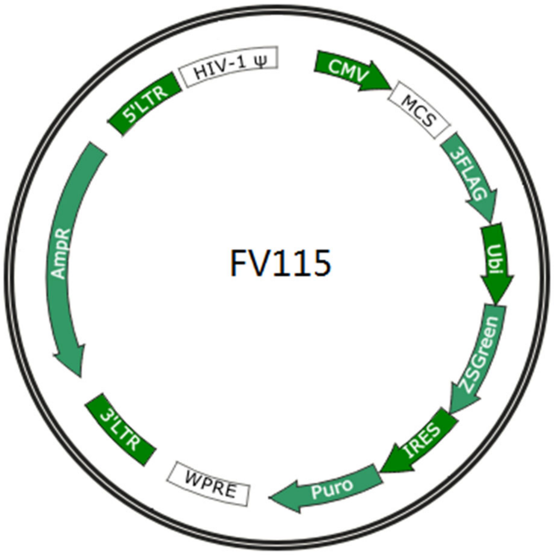

Figure.S1. The information of lentiviral vector FV115. Size: 10kb. Selection marker: ZSGreen/Puromycin. Frame structure: CMV-MCS-3Flag-Ubi-ZSGreen-IRES-Puromycin. MCS forward primer: 5'-CGCAAATGGGCGGTAGGCGTG-3', reverse primer: 5'-AGTCCCGTCCTAAAATGTC-3'. Restriction site: EcoRI and XbaI.

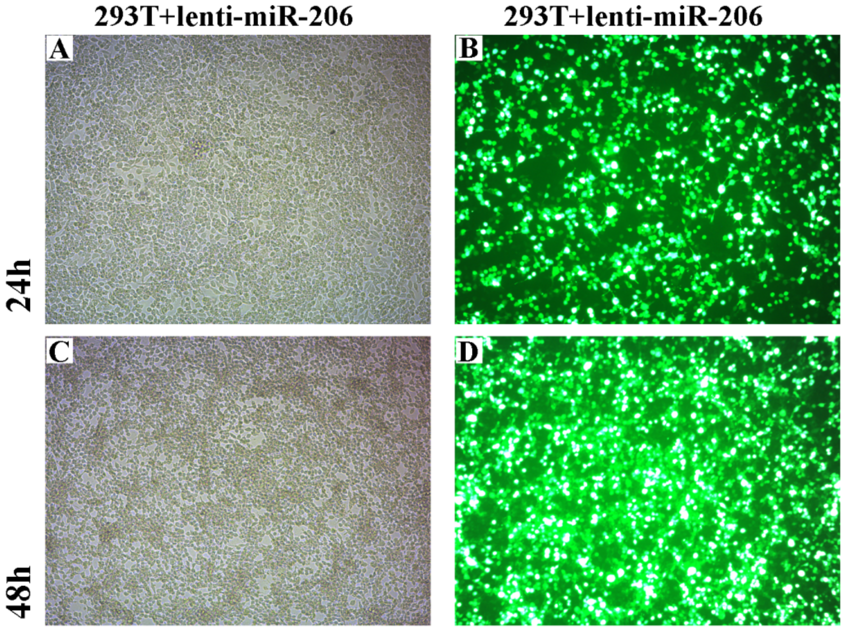

Figure.S2. Representative images of lentiviral miR-206 transfection with 293T cells. A and C: light microscopic image at 24h and 48h (100×). B and D: fluorescent microscopic image at 24h and 48h (100×).

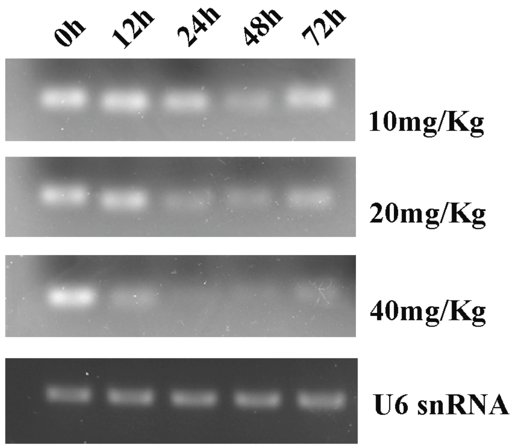

Figure. S3. Effect of miR-206 silencing on expression of miR-206 in koi carp skin. The koi carp weighing ~10g were received a tail-vein injection with different dose (10mg/kg, 20 mg/kg and 40 mg/kg), then detected at different time points using RT-PCR technique. The group conducted at 0h was taken as the control group and U6 sRNA expression was detected as the internal control.

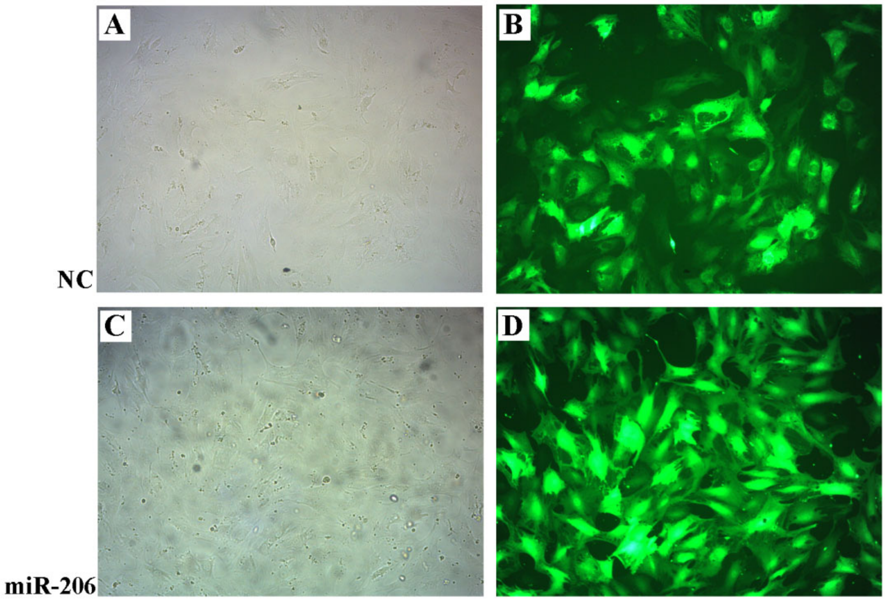

Figure. S4. GFP protein level of melanocytes transfect with lentivirus. A and C: light microscopic image of negative control (NC) and miR-206 sponge groups (100×). B and D: fluorescent microscopic image of negative control (NC) and miR-206 sponge group (100×).
