# Supplementary material for: MicroRNA-206 Regulation of Skin Pigmentation in Koi Carp (Cyprinus carpio L.)
Source: Front Genet. 2020 Feb 12;11:47. doi: 10.3389/fgene.2020.00047 (PMC7029398; doi:10.3389/fgene.2020.00047)
Supplement: Supplementary file 2 [file Table_1.docx]

**Table S1.** Primers for mRNAs and miRNAs quantitative in Koi carp

| Name | Sequence |
| --- | --- |
| miR-206 | GCGCGTGGAATGTAAGGAAGT |
| miR-181a-5p | GCGTCCTTCATTCCACCG |
| miR-217 | GCGCTACTGCATCAGGAACTG |
| miR-125b-5p | GCGTCCAGTTTTCCCAGGA |
| miR-196a | GCGCGCTAGGTAGTTTCATGTT |
| miR-125c | GCGTCCCTGAGACCCTAACT |
| Mc1r-F | AGGAATCTCCACTCGCCAA |
| Mc1r-R | GCCCGTGCTCCGTCAATAA |
| Tyr-F | CACGGTCTCCGATCTTCCC |
| Tyr-R | CATCACGCCAGTCCCAGTA |
| Dct-F | CCCTGCGTTCATCACCTG |
| Dct-R | CTGTCACACACCACTCCCC |
| U6-F | CTCGCTTCGGCAGCACA |
| U6-R | AACGCTTCACGAATTTGCGT |
| *β-actin*-F | CGTGATGGACTCTGGTGA |
| *β-actin*-R | ACAGTGTTGGCATACAGGT |
